# Advice from “pracademics” of how to apply ecological dynamics theory to practice design

**DOI:** 10.3389/fspor.2023.1192332

**Published:** 2023-05-24

**Authors:** Jia Yi Chow, Chris Button, Miriam Chang Yi Lee, Craig Morris, Richard Shuttleworth

**Affiliations:** ^1^ Physical Education and Sports Science, National institute of Education, Nanyang Technological University, Singapore, Singapore; ^2^ School of Physical Education, Sport and Exercise Sciences, Dunedin, New Zealand; ^3^ Sport Singapore, Singapore, Singapore; ^4^ Independent Researcher, London, United Kingdom

**Keywords:** ecological dynamics, practice design, pracademics, constraints-led approach, nonlinear pedagogy

## Abstract

There has been an increase interest in knowing and enacting pedagogical approaches such as the Constraints-led Approach (CLA) and Nonlinear Pedagogy (NLP) which are underpinned by Ecological Dynamics in recent years among practitioners. While there seems to be a perceived uptake of such pedagogical approaches that encourages exploratory learning and the development of individualised movement solutions, there are still concerns on how these pedagogical approaches are enacted on the ground. In this paper, we the authors, as “pracademics”, attempted to address some of the common concerns that we are aware of from our regular interactions with academics and practitioners. In brief, we highlighted some of the common challenges related to sense making concepts from Ecological Dynamics and building connections to practice. We stressed the need to invest time to think differently to create representative learning environment, rethink how assessment is to be done, finding a balance between theoretical jargon and practical application as well as intentionally situating coach development and support. We may not have all the answers, but we hope this paper could provide a useful starting point on how to apply Ecological Dynamics Theory to practice design.

## Introduction

In recent years, there has been increasing interest in pedagogical approaches that focus on exploratory learning and encouraging learners to find their own movement solutions where context plays a key role. Notably, the Constraints-led Approach (CLA) and Nonlinear Pedagogy (NLP) have garnered significant attention among academics and practitioners to explore their use in supporting skill development and adaptation (1, 2). These pedagogical approaches (mentioned above) are underpinned by the theoretical framework of Ecological Dynamics where there is an emphasis on examining and accounting for interaction among constraints to understand the emergence of movement skills (3). Not surprisingly, such ideas are intuitive to practitioners as features of “nonlinearity” during learning are easily identifiable. For example, there is non-proportional change in movement behaviours as a response to practice (i.e., sudden leaps or drops in performance), multiple ways of moving to achieve the same performance outcome, learners' responding to changes in constraints (e.g., task, performer or environmental) and the infusion of practice variability to support exploratory behaviours among learners (4, 5). With the science of skill acquisition evolving rapidly in today's digital world, practitioners seem keen to understand more about learning with reference to these pedagogical approaches to further develop their craft.

The research surrounding Ecological Dynamics has centred on describing and explaining how pedagogy can be enacted by practitioners like teachers and coaches to account for nonlinearity in the learning process. Indeed, empirical studies have tended to focus on the impact of such pedagogical approaches on teaching, coaching and learning (6–10). Nevertheless, there is a concurrent need to gather more insights on what practitioners really think and know about pedagogical approaches based on Ecological Dynamics. For example, what is the actual translation of these ideas on the ground? Do practitioners really understand and believe in such approaches or are they blindly adopting the latest research-informed ideas? Also, what are the perceived challenges that practitioners face while trying to enact such approaches? Some critics have claimed a reluctance exists amongst Ecological Dynamics proponents to tackle the “nitty-gritty” reality of sport coaching situations (11). However, it should also be acknowledged that there is a need for good quality longitudinal data to show efficacy of these approaches. Moreover, emergence of new data types would also add opportunities to develop the evidence base. Nevertheless, the challenges of examining the efficacy of CLA or NLP serves to illustrate the complexity inherent in an ED approach. We agree that we need to hear more from practitioners working at the “coalface” to investigate how well they understand and utilise such pedagogical approaches. One important resource in this regard is to gather insights from individuals who have both academic and practical experience, otherwise known as “pracademics” (12). Hence, our aim in this article was to solicit candid insight from various pracademics to help address common concerns about pedagogical approaches based on ecological dynamics.

### Brief overview of some of the approaches based on ecological dynamics

For the sake of clarity, Ecological Dynamics is underpinned by ideas from Ecological Psychology, Complexity Sciences and Dynamical Systems Theory. It recognises the continuous interaction between the mind, the subconscious control mechanisms of the body, and the environment in learning (13). Importantly, goal-directed behaviours emerge because of the interactions among constraints, and this is a key aspect of using ecological dynamics to understand the control and coordination of the human neurobiological system (1). From CLA, the emphasis is on examining the way task, performer and environmental constraints interact with one another to shape how degrees of freedom afforded to a learner (or even group of learners) are controlled for movement behaviours to be produced to attempt to meet the task goal. Learners are constrained to find their own individualised way of moving to satisfy the interacting constraints present in the performance environment (3). NLP provides specific pedagogical design principles that support an ecological dynamics perspective to encourage pedagogical practices to promote exploratory behaviours for learners to find individualised movement solutions. These pedagogical design principles are the provision of representative learning contexts, manipulation of constraints to shape behaviours, attunement to the impact of different informational constraints that focuses on either movement form or movement outcome, emphasis on task simplification to maintain perception-action coupling and the advantage of infusing practice variability to support exploration and exploitation (3, 14). Hence NLP is a broader pedagogical approach (than CLA) that also incorporates the key principle of manipulation of constraints to guide learners. The crux of both CLA and NLP is to provide a learning environment for learners to search and exploit opportunities for actions (affordances) that can be relevant for the way these movement behaviours can be used.

Nevertheless, while there has been a lot of interest in the use of CLA and NLP in enhancing teaching, coaching and learning, there remains many questions about how CLA and NLP can be delivered at the “coalface” (i.e., on the training field, practice gym, playing field, etc.). The ideas relating to Ecological Dynamics, while arguably intuitive, are not easy to grasp for many practitioners to apply directly to the practice environment. In many instances, practitioners may have challenges in how to design practices based on the pedagogical design principles of NLP (as an example) or to formalise how CLA would be manifested as it is not easy to map the kind of dynamic interactions that would occur (15). The anxiety of not being “in control” when designing practices and therefore not being 100% sure how learners would respond to interacting constraints are real. Also, the time needed to allow for exploration may not be a luxury that many practitioners have when syllabi are to be followed and where there are certain pre-conceived milestones or learning outcomes that need to be achieved. Some may also criticise that the amount of preparations needed to enact a CLA or NLP session could be huge and not easily managed on a regular basis (14). Specifically, in the cauldron of high-performance sport where the stakes of optimal athlete preparation are so high one can sympathise with a coach for “playing it safe” and sticking with tried and trusted learning approaches rather than dabbling in CLA or NLP. Thus, there are genuine concerns about the infusion of these pedagogical approaches and the impact that it may have on learners. In this paper, we want to provide some answers or thoughts to some of the above points raised and the difficulties in showing cause and effect of intervention on learning.

### Responses to questions

For this section, each of the authors of this paper provide their thoughts on some frequently asked questions about approaches based on Ecological Dynamics.

(1) What are the struggles that practitioners may have with pedagogical approaches based on Ecological Dynamics?
(2) Why are there these struggles with pedagogical approaches based on Ecological Dynamics?
(3) Where and how does the theory come in to help practitioners support their practice?
(4) Is there a place for theory in professional development for practitioners?
(5) What are the considerations to help practitioners enact pedagogical approaches based on Ecological Dynamics to support teaching and learning? Why are these important considerations?

First, we would provide a brief overview of the background of each of the contributors.

(1) Chris Button (CB)

Chris Button is a Professor of Motor Learning at the University of Otago, New Zealand. Chris has coached football at various levels (i.e., from children, to elite, through to masters) for over twenty years. Chris is also interested in water safety education and in recent years has undertaken numerous studies and interventions investigating how best to teach children transferable water safety skills and knowledge. Chris is lead author of the textbook: “Dynamics of Skill Acquisition: An Ecological Dynamics approach” (1).

(2) Jia Yi Chow (JYC)

Jia Yi Chow is a Physical Education teacher by training, and he is currently the Associate Dean for Programme & Student Development at the Office of Teacher Education, National Institute of Education, Nanyang Technological University, Singapore. Jia Yi mentors PE teachers for Singapore schools and prepares sports science undergraduate students for the sports industry. He enacts CLA and NLP regularly in his classes and works closely with key local stakeholders in the Ministry of Education and National Sports Institutes in Singapore. He also shares his work on a regular basis with international audiences at workshops and conferences.

(3) Miriam Lee (ML)

Miriam Lee is a Senior Manager at Sport Singapore, a national agency that promotes sports and physical activity. Miriam works with early childhood educators and sport coaches from the local community to develop the fundamental movement skills of preschool children. Her PhD research was on “Nonlinear Pedagogy and its application in Singapore Schools”, pursued at the National Institute of Education, Nanyang Technological University, Singapore. She continues to share and discuss ideas from Nonlinear Pedagogy through her interaction with practitioners and local stakeholders.

(4) Craig Morris (CM)

Craig Morris is an Olympic Canoe Slalom coach and high-performance coach/mentor based in London, UK. In recent years, Craig has begun to share his experiences of exploring and applying an Ecological Dynamics approach to sports coaching through academic journals and podcasts.

(5) Richard Shuttleworth (RS)

Richard Shuttleworth is the Coaching Director at Sport Singapore. He has experience leading and managing high performance development strategies, coaching and athlete development trajectories, team dynamics, in sport development and education systems in Europe, Asia and Australasia. He has also supported several successful Olympic sports campaigns at the Australian Institute of Sport (Australian Sports Commission) as Skill Acquisition Specialist spanning Beijing and London.

• What are the struggles that practitioners may have with pedagogical approaches based on Ecological Dynamics?

CB: Firstly, a challenge is to prioritise time to plan coaching sessions based on Ecological Dynamics (ED). As a coach, one should be very clear about the learning objectives of a session and how to integrate key pedagogical principles into a practice plan. I admit that I rarely spend sufficient time planning what to coach and thinking about how I should do it. Rather than follow a recipe rigidly (for practice design) I prefer to have a scaffold or some key activities which I build upon and fill in (with the learners) as the session unfolds. A common misconception is that an approach, like NLP, that is founded upon a principle like self-organisation can be done with minimal preparation. However, the more experience of coaching with ED I develop, the more I realise it is important to spend time thinking in advance how to best deliver the session, even if the product is usually different to what was planned. Similarly, once I have delivered a session, I need to more consistently note down what went well and what did not. I think I rely a little too much on memory and my perceptions for how things went, rather than systematically planning and reflecting on the process, successes and failures! Notably, one of the best opportunities for ED right now is to leverage developments in analytics, tech and performance analysis to develop systems and infrastructure to help with this.

Another struggle I continue to have is to develop strategies to involve the learner in the process of designing practice. In my work on water safety education for example, I have usually dictated what will be taught, where and when it will happen etc. I try to justify to myself that it is necessary to ensure the safety of my learners, but perhaps it is more that I am usually teaching children (approximately 7–11 years old) and I have not trusted them sufficiently to co-create an effective learning environment. I am always on the lookout for new ideas and ways to make coaching a genuinely reciprocal process and better engage the learners in practice design.

JYC: For me, the key struggles seem to be associated with understanding the language that is used pertaining to Ecological Dynamics. The term, “Ecological Dynamics”, can already be daunting for a practitioner who may not have any previous idea about complexity theories and how they may support skill acquisition. This in turn leads to a lack of proper understanding of how these pedagogical approaches work in the learning context. Of course, practitioners could still use these approaches but may not be able to fully utilise what these approaches could offer. Without a proper understanding of what it means from an ecological perspective, practitioners tend to copy examples of practices designed by other practitioners who purport the use of CLA or NLP. In most instances, such copy and paste could result in less success in supporting effective skill development, which in turn, leads to frustration on the part of the teacher or coach who is trying to use CLA or NLP. But that seems to be the typical grouse that a recipe should be provided to practitioners so that they can then replicate the lesson to achieve their desired learning outcomes for the learners. From my experiences with Student Teachers and In-service Teachers, there can be situations where they would want the “answers” to how to teach using a specific design principle but without considering that it is also situational as it can depend on the context of the learners on that day and the shift in learning outcomes for the session.

Another typical question that I get asked a lot is where the teaching of technical skills is important or if it should precede the incorporation of modified representative games in PE. My response is typically associated with what the practitioners want as a learning outcome: (i) to show the best form or (ii) to achieve the task goal in their own functional way. By asking this question, it typically sets the practitioner to start thinking about ways to achieve the outcome rather than be overly focused on the movement form (and thus an emphasis on technical skills). This inadvertently also leads to the issue of assessment and how practitioners share that they are sometimes constrained by assessment rubrics that emphasises the attainment of some expected movement form (especially in fundamental or foundational movement skills). Assessment shapes behaviours (and teaching behaviours) and thus an assessment that is focused on adaptability would be aligned to the design principles for NLP (as an example) rather than one that focuses on movement form replication.

ML: The first struggle that I often see practitioners face is absorbing and understanding the terminologies associated with Ecological Dynamics. Terms like “dynamical system theory”, “complex systems”, “nonlinearity”, “representativeness”, “affordances”, “information-movement coupling” and even “Ecological Dynamics” are jargons to the common folks not familiar with the literature. They often switch off once we start to introduce such terms, and more so when we try to explain it to them. It is always a challenge to balance between simplifying it for the layperson and without losing the essence of what these terms mean.

Another common struggle with practitioners working with young children is coming to terms with the notion that “there can be more than one way to achieve a task goal”. When it comes to teaching and learning gross motor skills or fundamental movement skills (FMS), there is a tendency still to hold on to traditional approaches in which the objective is primarily to achieve a predetermined criterion movement form. This is especially the case for sport coaches where practice sessions often involve drill-like and prescriptive instructions, despite efforts to share with them about exploratory pedagogy approaches.

As for the early childhood educators I work with, most seem open to ideas from Nonlinear Pedagogy such as letting children “explore and discover their own functional movement solutions”. Afterall, the preschool years are about exploration and discovery of themselves and the world around them. The part practitioners find hard to reconcile is the way a child's movement should be observed and assessed. Questions such as “If I don't use a movement checklist, then how do I know how well my child is progressing?” often emerge. On my part, I am constantly on a search to find alternative ways of observation/assessment aligned to pedagogical approaches based on ED, but it still has been a challenge to find tangible solutions that satisfies the needs of these practitioners.

CM: A struggle I have witnessed relates to answering the question of efficacy regarding an Ecological Dynamics approach in a society that values linear causality abstracted via metrics of progress and performance based on predetermined “fundamentals” of a sport. Metrics that are often sought in tasks undertaken in impoverished environments and/or supported by “evidencing” a growing knowledge about a specific technique, play or discipline through the spoken word. Amidst systems/cultures that prioritise hierarchies, prescription, control and predictability to forecast future success and showcase return on investment, an ED practitioner can face a struggle in aligning with what is seemingly valued by vast swathes of sporting domains.

Shaping affordances with integrity to a nonlinear approach is often a source of struggle for coaches. Designing tasks to explore new invitations for action without over constraining them, and thus losing representativeness, can be a real challenge. This requires the coach not only to have a clear understanding of theory but also to go through a process of “letting go”. This may involve letting go of answers they already have to leave multiple possibilities open that are likely more representative of the task itself during competition. Indeed, the use of “radical constraints” that narrow the field of affordances so much that perhaps only the solution the coach wants is available, is in my opinion little different to verbally solving a problem for them. We must ask ourselves are performers merely meeting the knowledge of the coach rather than exploring the thing itself? Perhaps, this is where they may learn that something works after exploration (e.g., experiencing success to meet task outcome). The understanding of why it works could come later upon reflection or further discourse with a mentor or peers.

RS: Coaches may have trouble learning about the use of constraints and CLA. It is a challenge to engage fully with “CLA”, but some coaches manage by “flirting” with the approach and applying a degree of pragmatism instead of “marrying” totally to it. Others suggest it gives them tools to explore within their practice but also a chance to understand why and how so that they can create their own applied frameworks. For example, a coach I know experienced his biggest breakthrough when he explained how ED opened his eyes to seeing the value in opportunities on a macro and micro level, thus helping him to create a new bottom-up individualised curriculum (needs-based solutions) for swimming. It helped guide the way he viewed how a child could optimise learning using an aquatic environment (i.e., macro level) to focus on saving lives from drowning and promote health. In this case, competitive swimming is therefore not representative of the task and more work was needed changing the environment and experiences. On a micro level in performance swimming, he was able to switch his attention from energy systems, and prescribed volume-based training to a skills focus. Both frameworks side by side looked similar but the difference was the intention. Training energy systems could neglect skill but training skill he assured me, also trains the energy systems. This could be an example of the knowledge of the environment, supporting better adaptation to the world around us and thereby optimising performance.

In addition, facilitation and guidance, practitioners can usually acquire strategies to transfer ED language into their own “action-based terms” and simplify (e.g., generally affordances become opportunities, self-organisation is adapting, attractor behaviour are tendencies etc. However, original meanings are usually lost in translation and can often be corrupted through cognitive biases).

• Why are there these struggles with pedagogical approaches based on Ecological Dynamics?

CB: Time is limited, and the digital world provides so much information for practitioners that the fundamentals of the process (like planning, reflection, co-creation, etc.) may be overlooked. Seductive concepts like life-hacks and You-Tube clips offer practitioners a plethora of potential shortcuts to bypass the dense academic literature and search for quick and ready answers to their problems. However, I tend to feel most satisfied as a coach if: (1) I have taken the time to take some notes before and after a practice session; (2) I have listened to learners and adapted my session to better suit their needs, and (3) myself and the learners were engaged, focussed and challenged by the activities we co-created. Perhaps because ED does not typically provide detailed instructions for coaching (a recipe book style), some may be challenged to put in the background work required to apply the principles successfully. This calls for incorporating interdisciplinary teams to support the use of such approaches.

JYC: There are probably a few reasons. It is possible that these terms may not have been explained properly to practitioners or in a way that is too technical that, without adequate prior knowledge about the theoretical concepts, could lead to confusion in understanding what CLA and NLP represents. Or, in other instances, the prior knowledge may be superficial (i.e., knowing the terms *per se* but not really what the concepts mean and how it would impact practice design) and thus there is a lack of clarity in how the ideas are utilised. In other occasions, the teachers or coaches may still follow quite a top-down or prescriptive perspective to how they think designing a practice could entail even though they may consider CLA and NLP as an intuitive approach to account for individualised learning. These practitioners may find it difficult to “not be in control” and allow for students to explore. Letting go of control would seem to be the hardest thing to do even though it seems to be the right thing in their mind. Thus, they may then resort to copying and pasting learning activities that they know worked when demonstrated by others.

ML: In my opinion, there are several reasons associated with these struggles. Firstly, it takes time to make sense, ask questions, try it out, but there is not always the luxury of time for this self-discovery process especially for practitioners who have competing demands. In some instances, academics continue to use abstract terms and examples to explain, making it hard for those not familiar with the language to understand and relate to. Most importantly, coaches and educators need practical examples to help them see how it is implemented and reassurance that ED pedagogical approaches work in practice for the early years. While online material such as FMS activity videos are readily available, many of these resources point towards learning FMS in isolation and fail to take into consideration the interaction that occurs between the learner, environment and task. Another reason why coaches may choose not to adopt ED approaches could be associated with the expectations from parents and others around, especially for paid programmes. For example, a coach once shared how he agreed with this approach, but he was hesitant to implement as parents may complain if it looked like he was not teaching and if the class looked messy. Perhaps, it would have helped if a clearer communication of the approach up front to the parents could clarify any misunderstanding on why the session was conducted in such a manner. Last of all, adopting an ED pedagogical approach requires a mindset shift for most practitioners, and if most of society do not embrace it, then it is probably easier for them to stick to old ways for the moment.

CM: The focus of study for the Ecological Dynamics practitioner is the reciprocity of the performer-environment interaction. Hence, attributing the efficacy of any one intervention or component of that interaction upon progress and performance in a causal linear fashion that is craved by many organisations is incompatible with the approach. Resultantly, the ED practitioner may find themselves unable to show cause and effect of their coaching in a way that is valued by their key stakeholders (club, programme, federation, parents etc). This can in turn leave them in a vulnerable position, often facing feedback regarding a perceived dereliction of duty or negligence by those who conceptualise the role of the coach in a starkly different manner. To exemplify, ecological approaches situate the role of the coach as an environment designer, problem setter and guide by the side, coaching interactions above actions. In contrast, the common societal conceptualisation of a coach is more as a gatekeeper of knowledge who disseminates expertise to performers from a hierarchical position through what and how of problem solving via technical models, set patterns and verbal instruction. Consequently, if progress is assessed in dualistic terms of performer as a separate entity to the environment, then the impact of an ED practitioner's work may well not meet expectations of their stakeholders.

RS: Applied sport coaching practice is often guided by a scientific approach, manifested in models or frameworks, which are viewed as the preferred or only way to facilitate athlete performance and development. This position can be problematic because the constitution of scientific knowledge has little meaning when attempts are made to apply it (i.e., a coaching approach) without consideration of context, people, and settings. It can be argued that such Ecological Dynamics frameworks for coaching have not been developed in partnership with coaching education, coaching process and applied practice design and as such are stand-alone models or frameworks that espouse an alternative approach to normal coaching and endure similar criticisms to more outdated approaches (i.e., new way vs. old way).

The issue is further exacerbated by the polarisation of athlete and coach learning, where all too often these two domains are reduced to separate entities evident in traditional coach education and athlete development practices. A concern with approaches (to coaching) is that what was once a good idea on how to describe and explain how skilled behaviour emerges can quickly become a coaching ideology where there exists a need to promote and defend a viewpoint/theory giving rise to a doctrine or a mutually exclusive approach claiming prominence. Ability to elicit the emergence of skilled behaviour over time risks being marginalised or even disregarded in favour of a new alternative. Rather, experiencing a buffet of learning principles that can be tasted and applied to coach-performer interactions in context (co-adapting menu).

• Where and how does the theory come in to help practitioners support their practice?

CB: Theory is the foundation upon which good practice is built. It provides a solid and reliable base upon which practitioners can design learning experiences. When it is most effective, theory sits in the background “unnoticed”, but practitioners can continually refer to it for guidance and reassurance. Powerful theoretical ideas such as self-organisation and affordances help practitioners to continually monitor and question their activities (and assumptions about learning).

JYC: In my view, there is a tendency for practitioners to use approaches that intuitively work for them or if they believe that the pedagogical practices used are “tried and tested”. Thus, they may be able to know the “what” or even “how” to teach/coach but may lack the understanding of the “why”. Is this important to know the “why” of what they do? I believe understanding why they design learning tasks in specific ways would give them greater adaptability in adjusting their practices to meet the needs of the learners. Consider the point on differentiated instructions where a practitioner can have the adaptability to skilfully manipulate constraints to challenge different learners in a class or practice session differently according to your competencies and needs. With a deeper knowledge of theory, a practitioner will then have more flexibility and understanding on how their practices can be designed purposefully.

ML: The theory provides a framework to guide practitioners to design and deliver practice sessions that are child-centred and meaningful for individual learners. Having a sound understanding of the theory will give practitioners a better appreciation of ED pedagogical approaches and to be in more control in varying situations: To be able to modify activities according to learners' needs, co-create activities, and to embrace unpredictability that occurs in learning.

CM: Theory has played a pivotal role in shaping “why I coach the way I coach” whilst also enhancing confidence in my approach. Furthermore, it has invited me to co-create this “why” with athletes, sharing our perceptions of what the sport is and reflecting upon how congruent our practice methodologies are in preparing for skilled performance in competition. In essence it is an anchor of reference for practitioners in the loop of “observe to design, design to observe”. As in an ED approach, a role of the coach may be guidance without specification toward information that could invite skilled action, so too theory guides the attention of the practitioner. For example, in my own practice, my attention is now guided toward affirmation of skilled adaptability by athletes, where once I saw movement variability as an error in the pursuit of consistency and repeatability. Theory therefore may be viewed as a companion or co-coach supporting our own decision making and development as we too seek to support the decision making and development of others.

RS: There exists a need for an experienced facilitator who can develop trust and a caring relationship that allows him/her to elicit a coach's existing perceptions, views, beliefs (i.e., intrinsic dynamics) and begin to question key assumptions in a safe, open yet uncertain environment. This is before any imparting of new knowledge which often will contrast and may even conflict with any stable self-held viewpoints and explanations of how human behaviour emerges [i.e., emergence (radical emergence) competing with the concept of design for learning foundational and skilled movement]. With practitioners, over time terminology can become normalised, easier to understand and more relevant to their ability to improve an individual's performance capabilities. If the practitioner possesses a natural interest and is motivated to pick up knowledge and acquire an understanding of ED, they become more motivated to establish deeper personal interpretations and meaningful connections rather than making mental representations and comparisons between approaches which is more often the case in coaching education pathways.

• Is there a place for theory in professional development for practitioners?

CB: Practitioners can operate and develop themselves in the absence of a theoretical model of the learner, indeed many have done that. However, “professional development” infers that practitioners are systematically preparing themselves to operate at a high level. In such a case then theory informs evidence-based practice and has a valuable place in a practitioner's toolbox. Whether it is coaching a junior football team or a national league futsal squad, I have found reassurance in my own professional development that has been informed by scientific theory.

JYC: There is certainly a place for theory in professional development for practitioners in my view. The above point about having theory support practice is key to enabling practitioners go beyond just trying to find “suitable” activity plans and re-enact them. Practitioners must go beyond the “copy and paste approach” of delivering practices as this would not adequately account for the dynamism that we would observe in all teaching and learning contexts where learner-environment mutuality is inherently present. It is important for practitioners to know why they are designing their practices in a specific manner.

ML: Yes, theory is an important part of professional development as it provides the basis for learning new skills and knowledge. Nonetheless, theory needs to be presented together with practical examples to help practitioners make sense of it and eventually apply their own context. As part of professional development, practitioners also become learners themselves during which learning takes place through interaction, adaptation, and co-creation of new knowledge between the facilitator and other learners in the environment. When practitioners can understand that they too are part of a larger system that involves the interaction of constraints, they will be in a much better place to apply theory into practice.

CM: In my experience, yes for both practitioners and performers. Theory acts as a constraint upon coaching practice, guiding attention from the infinite possibilities for practice design and interventions that can create a random experience that confuses athletes. With a shared theoretical approach practitioners and performers have a coordinated approach to why they practice the way they practice enabling co-adaption with an anchored reference point amidst what is inherently a dynamic journey.

RS: There is a place for theory, but some considerations need to be taken. Experience suggests it is likely not the terminology that is the issue but the simplification of the terminology that needs careful attention. Understanding IN action makes for coaching IN moment (e.g., immersion or entanglement) and coaching the performer (not the curriculum/learning outcomes) a more authentic (i.e., coach interaction fidelity) transformational experience when supporting a coach in navigating their development journey through a particularly unforgiving paradoxical landscape (e.g., learning and performance exclusivity or mutuality).

• What are the considerations to help practitioners enact pedagogical approaches based on Ecological Dynamics to support teaching and learning? Why are these important considerations?

CB: Learning can and does happen, with or without, practitioners. A key consideration for practitioners is their curiosity or appetite to “understand” and thereby potentially facilitate the learning process. Practitioners can choose to be passengers (i.e., passive) in this journey of understanding or they can equip themselves to augment the trip and become active partners alongside the learner/s. In my experience, important considerations are the extent to which the skills that are practiced are retained over time, are transferable to other contexts, and finally are stable in the face of a change in constraints. Personally, I think Ecological Dynamics is unique (in contrast to other theories of motor learning) in its ability to support each of these considerations when co-designing and reflecting upon practice. In addition, we could also incorporate the use of technology to better capture the learning experiences, which can show the value of an ED approach.

JYC: The biggest challenge is the fear of “letting go” on the part of the practitioner. There is typically a strong desire to prescribe how learning should exactly occur for the learner which leads to a teacher-centred approach. The practitioner prescribes instructions that dictates the expected movement form expected from learners without accounting for individual differences in the learning context. There is a need to be the holder of knowledge and experience on how learning should be undertaken. Thus, it would need a mindset change on the part of the practitioner to be “comfortable being uncomfortable” to let learners explore their own movement solutions to accomplish task goals set out in the session. Without holding prescriptive informational constraints to tell learners exactly how to solve a movement problem is something that practitioners may need some getting used to.

ML: To bring practitioners along with us on the journey, we need to help them relate to ED pedagogical approaches in the first place. For example, by using language that the target practitioners can identify with, we may be able to unlock their interest and open the door for further conversations and discovery of a deeper understanding of this approach. Besides the practitioner, others in the ecosystem also need to be educated about this approach. They need to understand and be aware about the nonlinearity that may emerge during practice sessions. Parents, senior management, and others in society need to be on the same page and support this approach for practitioners to successfully implement it.

CM: Support on the journey toward an embracement of uncertainty, of letting go of “knowing” and the idea of controlling what we can control in favour of immersing ourselves in the actual goings on of things. This may not be a shift of simply one individual but may indeed be an entire organisation, as constraints of the system act upon a practitioner (e.g., viewing coach as a hierarchical position and prescribing learning) and can therefore inhibit invitations to act in a way that supports an ED approach. To exemplify, coaches need support to pacify a perception that they need to have a prescribed plan for practice design and that it needs to be right first time. As with athletes, coaches must be given and give themselves permission to explore, to iterate, and to understand that development is nonlinear and thus what works in one context at one time may not necessarily work on repeat.

Secondly, sharing the journey into ED is in my opinion critical. Not in a sense of taking people with you but rather co-creating and adapting on a direction of travel. It is in my experience important that practitioners remain authentic when considering a pedagogical shift. A big part of this is to share “why” with performers and colleagues and invite feedback and challenge. Indeed, without explanation such approaches can be viewed incorrectly as “hands off”. Hence, we must acknowledge that an ED approach is significantly different to mainstream conceptualisations of coaching and therefore if enacted without explanation of why to stakeholders, then we are likely to be swimming against the tide.

RS: While ED can be challenging to one's view of the world, it also allows for thoughts to be dynamic, as opposed to being fixed, explicit or requiring a process. Some coaches find this freedom of thought both in and outside of sport and coaching context to be self-empowering and helps them make deeper connections to the wider world and sense making. A surfer finds that each wave is different, and they can only act upon what the wave offers. Therefore, a mutual respect between them and the environment is created. That said, the coach is aware that when they coach sport and they are outside of their comfort zone, they resort to the explicit direct approaches to reassure themselves that they are missing anything. Repeating the technical knowledge to show apparent competency. Within the realm of exploring ED principles of learning, coaches can become more relaxed in their approach and recognise the “knowledge about” has now been replaced by their search to discover the knowledge (information within) of, just like the waves taught (invited) the coach which parts to surf.

### Further implications and considerations for the adoption of pedagogical approaches based on ecological dynamics

Based on the input and candid sharing by the co-authors of this paper, we hope the discussion has provided helpful insights to the reality of practising pedagogical approaches based on ED and certainly the challenges that we (and you may) have faced as well.

#### Investment of time, thinking differently and creating representative learning designs

One common theme has been the necessity of investment of time for forethought, planning and co-creation of practice. In a digital world where quick solutions are available “at our fingertips” this investment of time is a real, tangible barrier that may force many practitioners to shy away from these approaches. Also, we have identified that an “open mind” is required from practitioners to expose themselves to theory, to try new things, and to emphasise adaptability of skill-learning are crucial elements of successfully utilising such approaches. Undoubtedly, ED forces practitioners to think quite differently about the learner and the learning process and we acknowledge that it takes some courage and patience to turn away from strategies that may have remained unchallenged for decades. Another theme that emerged from many of our answers was the idea of how to best represent performance characteristics in practice environments. Whilst the concept of representative design was introduced quite some time ago (i.e. (16), it has only quite recently made a long overdue entrance into the motor learning literature (17). As practitioner's ability to modify and simulate learning environments continues to grow rapidly, this is one issue which will capture our attention in the years ahead.

#### More than just manipulating constraints

CLA is a framework that describes how humans self-organise movement solutions through interaction with the environment. Therefore, technically all coaching is constraints led. This does not mean of course that it is informed and anchored by a theoretical framework that aids a coach's observation, decision making and correspondence. In an Ecological Dynamics approach to sports coaching, it is the key principles of a Nonlinear Pedagogy that underpin a CLA, informing the decisions we make as coaches on the why, what, when and how of constraint manipulations (3). Principles such as representative learning design, repetition without repetition and perception-action coupling guide skilful use of constraint manipulation which enhances the chances of development and transfer to competition. Designing practice environments therefore is not about manipulation to guide performers to the coaches pre-determined solution but to ease the grip on preconceived ideas to pay genuine attention to the individuals search for solutions (18). This process is challenging when we genuinely want people to progress and the temptation to shortcut through heavy signposting to solutions can be strong. Here an understanding of underpinning theory can give a coach confidence, opening one up to new possibilities for action through the creative exploration of performers. Afterall sport can only move forward if we stop simply trying to replicate what has gone before.

#### Let us find a balance between theoretical jargon and practical application

But there is certainly a need to find some balance between theoretical jargon and practical application of knowledge. Ecological (psychology) and dynamical (systems) evolutionary language describing Ecological Dynamics is often a confronting barrier for curious coaches to explore further. This can be exemplified through futile attempts to link coach-performer interactions to relevant theoretical ideas and underpinning principles unless there is an experienced theoretically informed practitioner (e.g., pracademic) or coach developer.

Coaches have suggested simplifying the language and helping them to find a grounding position to see the landscape in front of them. For example, a coach only saw complex pieces of the swimmers’ physiological demands and the complex scientific needs. However, when he scaled back his thinking and looked carefully at the landscape of pool affordances, it became clear that the sport required very simple demands of reducing drag and increasing propulsion. From here he then built up his philosophy and appreciation of the skills needed to be performed under physical and psychological pressure.

#### But what about assessment?

Methodologies of assessment of an approach must be congruent with the approach itself, namely how the performer interacts with the environment that is representative of the performance context. Such assessments are inherently complex and rarely offer the certainty desired by those conducting them hence ED practitioners are often judged by results only. Given a nonlinear approach is a performer centred, long term approach to skill development, then in certain climates this gives the coach little time if results are not considered adequate in the short term (14). Thus, coaches can be left feeling vulnerable, isolated and without the relevant permissions to explore. It is possible they may lose their job before the impact of their work has time to show or perhaps the perceived needs of the coach to conform to pervasive societal views of what a coach is and what a coach does may begin to dominate their practice above the needs of the performer(s). A range of assessment methods that acknowledge exploration, sensitivity to affordances, and adaptation to constraints are much needed for the practitioner's toolbox.

#### Situated coach development and support

As ED positions performer (and coach) performance and development through the conceptualisation of an ecological learning system (19), where performance problems and subsequent solutions emerge from the moment-to-moment interactions between coaches, athletes, scientific principles, and contexts. Therefore, learning is not assumed to occur based on a predetermined model or framework, rather, learning is situated within dynamic, context dependent, and lived experiences of athletes' and coaches (20). Coaching, and therefore athlete learning happens *in* the moment, to elicit more contextualised, evolving, and adaptable interactions related to context. Rather than through a model or framework that has a definite start and end point (e.g., *plan, do, review*), reliant on memory-based post event reflections that can place the coach under pressure (e.g., peer, leadership, parental, and organisational etc) to appease apparent perceived need for more established traditional practice methods of learning and performance outcomes. The constellation of key actors in an ecological learning system can support the contextualisation and individualisation of athlete, coach, learning and development. Importantly, there could be greater emphasis on supporting coach education centred around acquiring knowledge of the environment (direct perception). Approaches based on Ecological Dynamics can promote continuous self-regulation of learning on the part of the coach and teacher so that the practitioner can explore and attain a better fit with the performance environment (21).

## Conclusion

We realise that many of you might also find yourself disappointed if you expected a detailed recipe of how CLA or NLP can be enacted from this paper. However, as we have explained, such pre-determined and mechanistic solutions are counter to the theoretical assumptions that underpin the ED approach. Instead, our aim was to encourage you to shift your mindset (away from concepts like prescription and standardization) towards practices that emphasise and support adaptability. Finally, we humbly recognise that we do not have all the answers to many of the important questions that have emerged with the development and uptake of these ED-informed approaches. It is indeed an on-going journey for us all as we continue to embrace exploration in teaching, coaching and learning.

## Data Availability

The original contributions presented in the study are included in the article, further inquiries can be directed to the corresponding author.
